# Specialized gray matter segmentation via a generative adversarial network: application on brain white matter hyperintensities classification

**DOI:** 10.3389/fnins.2024.1416174

**Published:** 2024-09-30

**Authors:** Mahdi Bashiri Bawil, Mousa Shamsi, Abolhassan Shakeri Bavil, Sebelan Danishvar

**Affiliations:** ^1^Biomedical Engineering Faculty, Sahand University of Technology, Tabriz, Iran; ^2^Radiology Department, Tabriz University of Medical Sciences, Tabriz, Iran; ^3^Brunel University London, London, United Kingdom

**Keywords:** gray matter segmentation, deep learning, conditional generative adversarial network, white matter hyperintensities, juxtacortical WMH, WMH classification, MRI images, multiple sclerosis

## Abstract

**Background:**

White matter hyperintensities (WMH) observed in T2 fluid-attenuated inversion recovery (FLAIR) images have emerged as potential markers of neurodegenerative diseases like Multiple Sclerosis (MS). Lacking comprehensive automated WMH classification systems in current research, there is a need to develop accurate detection and classification methods for WMH that will benefit the diagnosis and monitoring of brain diseases.

**Objective:**

Juxtacortical WMH (JCWMH) is a less explored subtype of WMH, primarily due to the hard definition of the cortex in FLAIR images, which is escalated by the presence of lesions to obtain appropriate gray matter (GM) masks.

**Methods:**

In this study, we present a method to perform a specialized GM segmentation developed for the classification of WMH, especially JCWMH. Using T1 and FLAIR images, we propose a pipeline to integrate masks of white matter, cerebrospinal fluid, ventricles, and WMH to create a unique mask to refine the primary GM map. Subsequently, we utilize this pipeline to generate paired data for training a conditional generative adversarial network (cGAN) to substitute the pipeline and reduce the inputs to only FLAIR images. The classification of WMH is then based on the distances between WMH and ventricular and GM masks. Due to the lack of multi-class labeled WMH datasets and the need for extensive data for training deep learning models, we attempted to collect a large local dataset and manually segment and label some data for WMH and ventricles.

**Results:**

In JCWMH classification, the proposed method exhibited a Dice similarity coefficient, precision, and sensitivity of 0.76, 0.69, and 0.84, respectively. With values of 0.66, 0.55, and 0.81, the proposed method clearly outperformed the approach commonly used in the literature, which uses extracted GM masks from registered T1 images on FLAIR.

**Conclusion:**

After training, the method proves its efficiency by providing results in less than one second. In contrast, the usual approach would require at least two minutes for registration and segmentation alone. The proposed method is automated and fast and requires no initialization as it works exclusively with FLAIR images. Such innovative methods will undoubtedly facilitate accurate and meaningful analysis of WMH in clinical practice by reducing complexity and increasing efficiency.

## 1 Introduction

White matter hyperintensities (WMH) or white matter signal abnormalities observed in MRI results have a significant impact on the diagnosis and monitoring of brain diseases (Fazekas et al., 1987). T2 fluid-attenuated inversion recovery (FLAIR) images offer the most effective visualization of WMH for clinical application (Wardlaw et al., 2013). Consequently, WMH observed in FLAIR images have emerged as potential markers for neurodegenerative diseases such as cerebral small vessel disease (SVD) and multiple sclerosis (MS) (Kuchcinski and Wright, 2021). Since the premier research on brain abnormality (Fazekas et al., 1987), WMH have been commonly categorized into two primary classes: periventricular WMH (PEWMH) and deep WMH (DWMH) (DeCarli et al., 2005). However, it is important to note that other significant subclasses such as juxtacortical WMH (JCWMH) also exist (Kim et al., 2008), but these receive comparatively less attention. Accurately detecting and classifying WMH into meaningful categories not only aids in disease diagnosis but also facilitates the identification of various cognitive, microstructural, and clinical correlations (Griffanti et al., 2018).

Manual segmentation of white matter hyperintensities (WMH) is known to be a laborious task that can be subject to intra- and inter-observer variabilities (Tran et al., 2022). Conversely, automated segmentation methods have the potential to streamline clinical processes. A variety of accurate WMH segmentation techniques currently exist, ranging from statistical to machine learning algorithms (Shah et al., 2023; Huang et al., 2023). Some of these methods are tailored to address specific diseases such as SVD or MS by co-analyzing the ventricular structures of the brain and other normal tissues (i.e., gray matter) (Tran et al., 2022; Atlason et al., 2022; Rieu et al., 2021). In recent years, AI applications in medicine have surged, particularly in medical imaging. Deep Learning (DL) has been especially successful in tasks like image processing and segmentation, potentially enhancing or even surpassing human diagnostic accuracy (Borys et al., 2023; Hosny et al., 2024; Kassem et al., 2023; Kumar et al., 2023a, b).

Following the development of powerful WMH segmentation methods, there is a practical need to localize and classify WMH to investigate their correlation with the development or progression of brain diseases ranging from Alzheimer’s to MS (Parent et al., 2023). Furthermore, to have distinct classes with different clinical risk factors, WMH could be classified into specific progressions based on features such as location, volume, and contrast with surrounding normal-appearing tissue: (1) multiple, small, low contrast lesions in DWMH (2) large, confluent lesions in PEWMH (3) high contrast lesions confined to juxtaventricular WMH (Jung et al., 2021). The integration of machine learning algorithms has enabled the redefinition of the Fazekas scale for automatic assessment, alongside deep-learning-based detection and measurement of WMH and ventricular analysis (Rieu et al., 2023; Hong et al., 2020). While comprehensive automatic WMH classification is currently missing in state-of-the-art research on brain diseases, WMH can be meticulously categorized into subgroups such as subcortical spots, peri-basal ganglia, anterior, and posterior subcortical patches so that the impact of different WMH can be analyzed on cognitive impairments (Wang et al., 2022; Yang et al., 2023; Guo and Shi, 2022; Melazzini et al., 2021). In another study, WMH were manually divided into five distinct classes: deep frontal, periventricular, juxtacortical, parietal, and posterior with juxtacortical, deep frontal, and parietal WMH being linked to cognitive impairment, while the others showed no association with cognitive decline (Phuah et al., 2022).

During neurodegenerative diseases, both white matter (WM) and gray matter (GM) are affected by demyelination (Nakamura and Fisher, 2009). Advanced MRI techniques have enabled direct visualization of GM atrophy and demyelinating lesions, that progress over time and in the early stages of MS (Honce, 2013). Also, studying gray matter (GM) meticulously can uncover long-term neurological effects of mild COVID-19 infection. Individuals who tested positive for COVID-19 exhibited increased brain and GM shrinkage, as well as tissue damage, compared to a control group (Kumar et al., 2023a, b). Therefore, the study of GM-related demyelination plays an indisputable role in the pathology of neurodegenerative diseases. JCWMH fit best in GM studies. For instance, fronto-parietal and temporal PEWMH and have been independently linked to the processing speed and episodic memory, respectively (Jiménez-Balado et al., 2022). In Jiménez-Balado et al. (2022), GM maps were first derived from T1-weighted images, and then JCWMH were identified based on the proximity to registered GM maps on FLAIR images. Needless to say, GM segmentation of a brain with abnormalities presents challenges arising from lesions and brain atrophy (Nakamura and Fisher, 2009). More specifically, the presence of lesions or brain atrophy disrupt the normal appearing tissue both in intensity values and borders. For instance, hypointense appearing lesions in T1 images have intensities close enough to GM tissue to be falsely segmented as GM by most common methods. Consequently, new algorithms have been proposed to enhance standard GM segmentation methods for abnormal images. Both earlier and recent studies such as Nakamura and Fisher (2009) and Zhu et al. (2022) have used T1 images to indirectly specify GM on FLAIR images for JCWMH classification. Of course, another approach to address this issue involves focusing on hypointensities in T1 space rather than hyperintensities in FLAIR space. In this case, the GM mask would be assuredly extracted, which allow a simple definition of hypointensities near cortical (Dadar et al., 2019). However, it is important to note that all hypointensities (i.e., black holes) are a subset of hyperintensities. Therefore, on the exclusive use of hypointensities visible in T1 images may not capture all JCWMH instances. In addition, the accuracy of well-established GM segmentation methods, including widely used tools such as FreeSurfer (Fischl, 2012), may not be as accurate as expected due to the overlapping intensities of black holes and GM in T1-weighted images (Dadar et al., 2021).

To reinstate the aforementioned points, the majority of studies have opted to utilize T1-weighted images for GM segmentation as they provide better contrast, although two key issues have been overlooked. First, the registration of T1 images on FLAIR is not flawless, even with the best algorithms, and factors such as patient movements and artifacts can interfere with the alignment between T1 and FLAIR images, resulting in imperfect registration. Second, the presence of hypointensities or hyperintensities overlapping with GM poses a challenge even for the most advanced GM segmentation methods. Thus, to the best of our knowledge, there is currently no specialized GM segmentation method for lesion-present MRI images that exclusively utilizes FLAIR images to identify JCWMH. In this study, we introduce a novel pipeline that aims to improve GM segmentation in the presence of lesions by using both FLAIR and T1 images to classify WMH, with a focus on JCWMH. The proposed pipeline starts by segmenting brain tissue using a well-established tool and applies basic morphological operations to create relevant tissue masks. Subsequently, it utilizes the extracted WMH and ventricle masks to create separate dilated masks through morphological operations. Finally, the processed GM mask is refined with a combined mask of all processed tissue masks. We employ this pipeline to generate specialized GM masks for both healthy individuals and MS patients from our extensive local dataset, in order to train a deep learning network such as a conditional generative adversarial network (cGAN). This model is trained to produce specialized GM masks based on FLAIR images, thereby reducing the dependency on T1 images, streamlining the complexity of the proposed pipeline and performing real-time. By generating specialized GM masks in conjunction with extracted ventricular masks, we classify WMH into three classes: periventricular (i.e., PEWMH), paraventricular (i.e., PAWMH), and juxtacortical (i.e., JCWMH) based on their proximity to the masks.

The first section of this paper reviewed existing literature and encountered obstacles. The subsequent sections are organized as such: section 2 will outline the proposed method, whereas section 3 will present its performance and outcomes. Sections 4 and 5 will discuss the findings and draw a conclusion, respectively.

## 2 Materials and methods

### 2.1 Subjects and MRI data description

The data of the article was drawn out of 1,000 healthy individuals and 270 MS patients imaged by a 1.5-Tesla, TOSHIBA Vantage scanner (Canon Medical Systems, Japan) at the Golghasht Medical Imaging Center, Tabriz, Iran. Standardized protocols for MRI scanning were followed, which included the use of various sequences such as [repetition time (TR) = 540 ms, echo time (TE) = 15 ms, flip angle (FA) =70°, field of view (FOV) = 230 × 230 mm^2^, number of slices = 18, acquisition matrix = (0, 256, 176, 0), voxel size = 0.45 × 0.45, slice thickness = 6 mm], T2-weighted sequence [TR = 4,800 ms, TE = 105 ms, FA = 90°, FOV = 230 × 230 mm^2^, number of slices = 20, acquisition matrix = (0, 352, 256, 0), voxel size = 0.33 × 0.33, slice thickness = 5 mm], T2-FLAIR sequence [TR = 10,000 ms, TE = 100 ms, inversion time (TI) = 2,500 ms, FA = 90°, FOV = 230 × 230 mm^2^, number of slices = 20, acquisition matrix = (0, 256, 192, 0), voxel size = 0.9 × 0.9, slice thickness = 6 mm], and diffusion-weighted sequence [TR = 2,585 ms, TE = 100 ms, FA = 90°, FOV = 230 × 230 mm^2^, number of slices = 15, acquisition matrix = (144, 0, 0, 144), voxel size = 0.8 × 0.8, slice thickness = 6 mm]. A neuroradiologist reviewed every scan of patients.

In this study, we employed the T2-FLAIR and T1 images acquired horizontally with the voxel sizes of (0.9, 0.9, 6) and (0.45, 0.45, 6) millimeters, respectively. Ethical approval was granted by the Tabriz University of Medical Sciences Research Ethics Committee, and written approval letters were obtained from all participating patients. Additionally, all raw data has been anonymized at the very first stage so that patients are trackable only by a patient ID not their name.

### 2.2 Manual WMH and ventricles segmentation

In order to quantitatively evaluate the performance of the proposed method, we randomly selected MRI data from 9 MS patients. Among these patients, there were four males (aged 30–59 years, mean = 43, SD = 14.7) and five females (aged 29–46 years, mean = 37.2, SD = 9.1). The manual segmentation and labeling of this MRI data was carried out by a radiologist with over 20 years of experience in assessing MRI scans. The image computing platform 3D Slicer (Fedorov et al., 2012) was used for the segmentation tasks.

**Figure 1:**
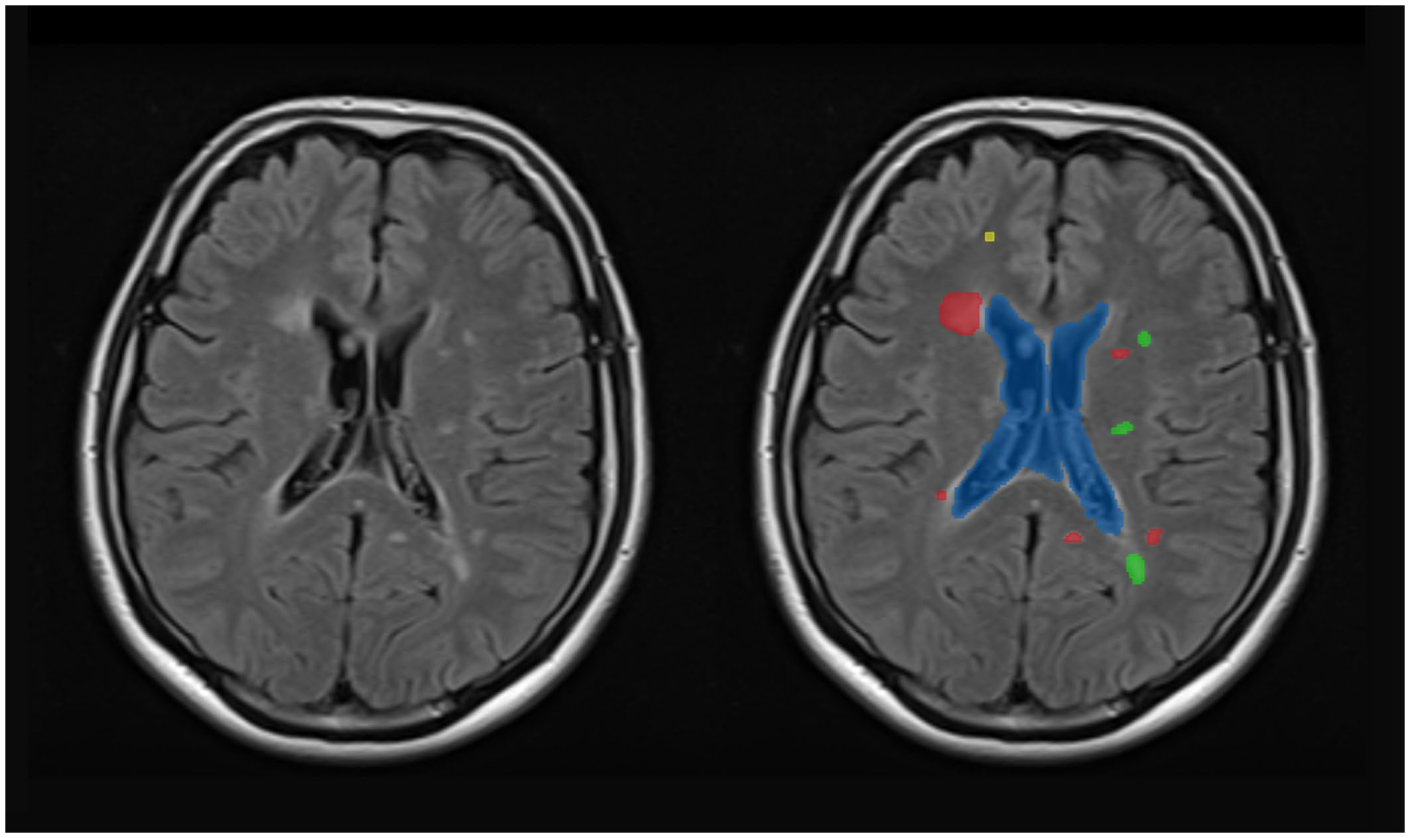
A sample slice of FLAIR images (left) and manually segmented WMH and ventricular system (right). The ventricular system is highlighted as blue, whereas the periventricular, paraventricular, and juxtacortical WMH are differentiated by red, green, and yellow colors, respectively.

**Figure 2:**
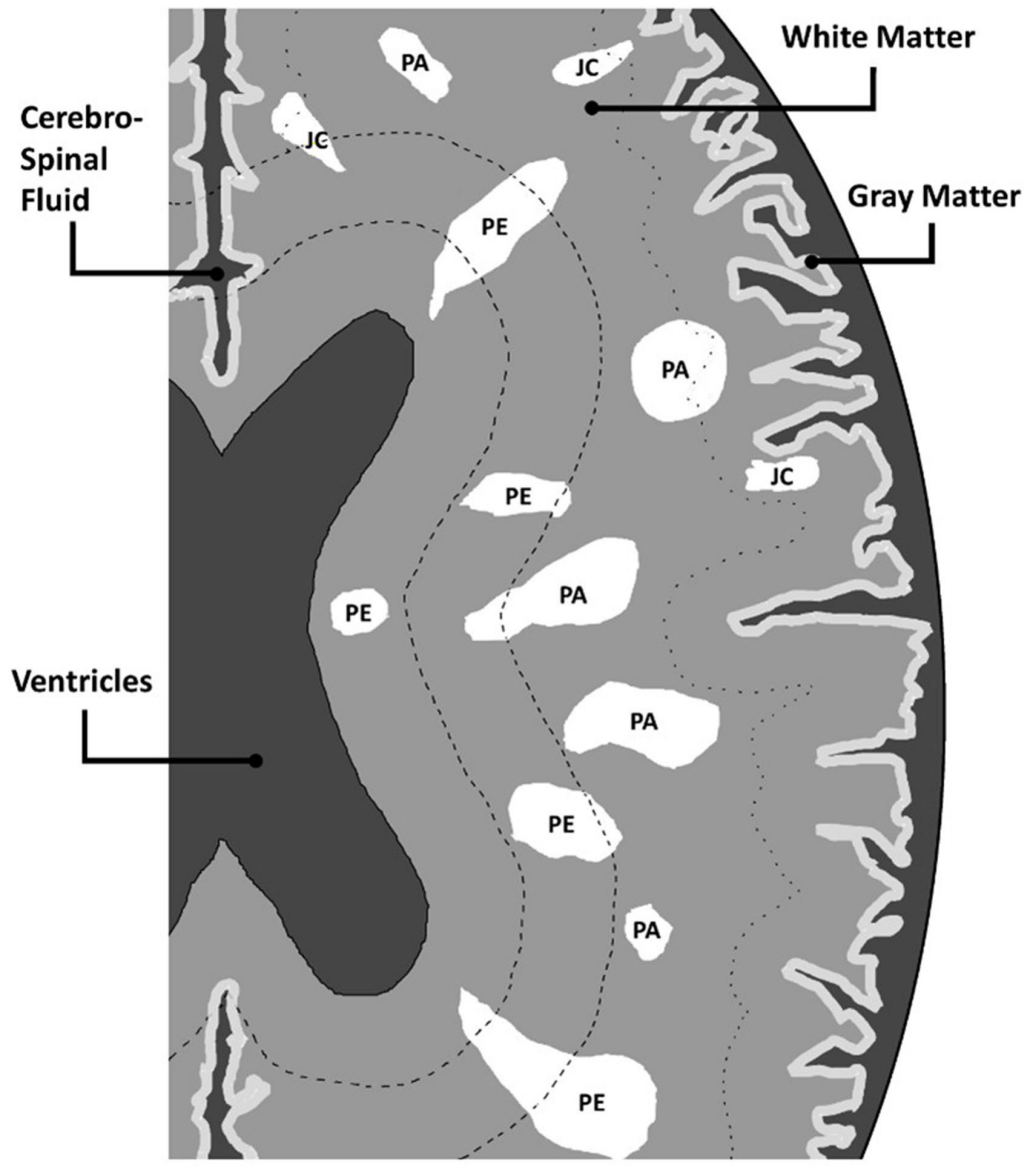
An illustration of WMH and corresponding labels, in a FLAIR slice. The abbreviations PE, PA, and JC are used to denote PEWMH, PAWMH, and JCWMH, respectively.

When segmenting WMH in the images, they were segmented into PEWMH, PAWMH, and JCWMH classes based on their distance to the adjacent ventricles and GM tissue. Figure 1 shows an example of a FLAIR slice of a patient, along with its manual segmentation and labeling. There are several rules that help classify WMH into above categories. These rules include continuity to the ventricles (Fazekas et al., 1987; Fazekas et al., 1993; van den Heuvel et al., 2006) or a distance of 10 mm from the ventricles (DeCarli et al., 2005) to determine whether the WMH is periventricular or deep, and a distance of 3–13 mm (Kim et al., 2008) to consider a new juxtaventricular category in addition to periventricular and deep WMH categories. Juxtacortical WMH can be characterized as small lesions, no more than 5 mm in diameter, located relatively close to the cortex, especially at a distance of less than 10 mm from the corticomedullary junction (Shan et al., 2017). The process of manual segmentation and labeling in this study followed Algorithm 1. To illustrate how the labeling process is implemented according to the algorithm, Figure 2 presents an illustration of a FLAIR slice depicting the 5 mm and 10 mm boundary of the ventricles, as well as the 5 mm boundary of the GM tissue.

### 2.3 Proposed pipeline

#### 2.3.1 Brain tissue segmentation and post-processing

In this study, we first register T1 images to FLAIR images using the *flirt command* of FSL (Jenkinson et al., 2012). The registered images are then segmented using the SPM toolbox (Friston and Penny, 2003) within MATLAB software^1^ to obtain three main masks of brain tissue in FLAIR space. However, it is challenging to obtain exact brain slices in both T1 and FLAIR images of a patient. Consequently, using the registered T1 images for brain tissue segmentation may result in less precise maps, especially for GM.

##### Algorithm 1 Decision-making process of WMH classification into three classes.

**Table:** 

For each WMH found: Calculate the distances from nearby ventricle and GM masks If the shortest distance of the WMH from the nearby ventricle ≤5 mm: It is a PEWMH Continue to line 1 If the mass center distance of the WMH from the nearby ventricle ≤10 mm: It is a PEWMH Continue to line 1 If the shortest distance of the WMH from the nearby GM mask <5 mm and the area of the WMH <20 mm^2^: It is a JCWMH Continue to line 1 Else: It is a PAWMH Continue to line 1

**Figure 3:**
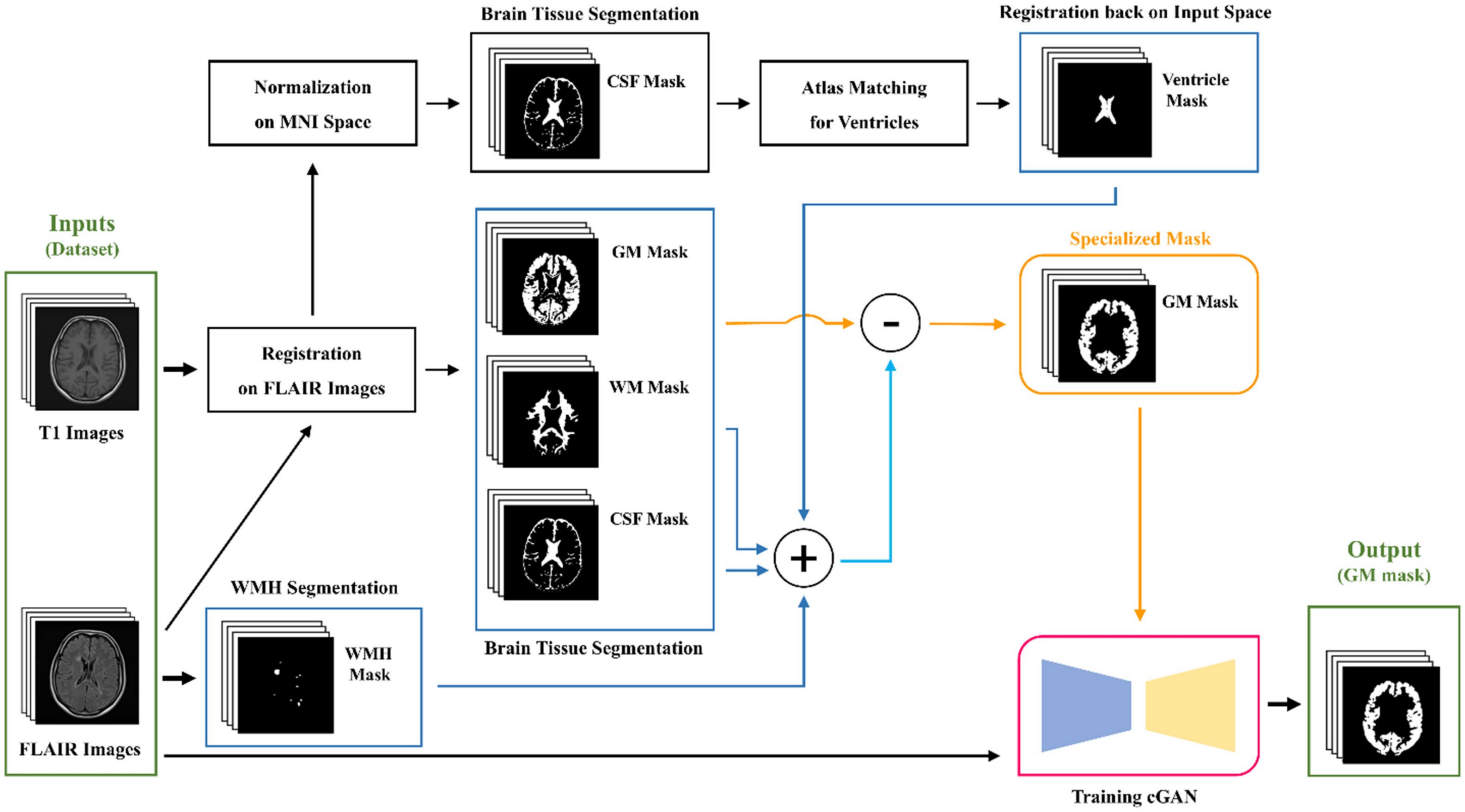
Graphical flowchart of the proposed method for the specialized GM segmentation pipeline and training a cGAN model.

To overcome this challenge, after segmenting the brain tissue with the SPM toolbox, we first attempt a few fundamental image processing morphology operations as a post-processing step to enhance the results obtained from the SPM. For the GM map, we use a low threshold to create a binarized GM mask that contains as many GM voxels as possible, while minimizing the inclusion of indeterminate voxels. For the WM mask, we use a high threshold to secure the most certain WM voxels. The CSF map, on the other hand, is post-processed similarly to the WM map, but with a lower threshold, as the accuracy of the CSF map is higher due to its distinct nature. All these post-processing steps are part of the brain tissue segmentation process outlined in Figure 3, which summarizes the key steps of the proposed pipeline for specialized GM segmentation.

#### 2.3.2 WMH and ventricles segmentation and post-processing

While LST-LPA (Schmidt, 2017; Schmidt et al., 2012) is utilized for WMH segmentation, a more intricate approach is taken for ventricular segmentation. It involves normalizing previously registered T1 images to MNI space, segmenting the normalized images to obtain CSF masks, and then matching these masks with MNI ventricular masks to identify potential ventricular regions. Subsequently, the ventricular masks are registered back to the input space, which is FLAIR. As in the post-processing steps for brain tissue maps, we employ fundamental morphological operations such as dilation, closing, and opening to the segmented WMH and ventricular masks. However, in this particular section, our main objective is to inflate the WMH and ventricle masks. The slight inflation of WMH masks is necessary due to the uncertain boundaries of segmented WMH, which can be done automatically or manually. To address this problem, we apply a 5-by-5 rectangular element. On the other hand, the ventricular masks not only face the same problem, but also have neighboring regions that may be falsely labeled or segmented as GM tissue. This is undesirable when studying the classification of juxtacortical WMH. Thus, Therefore, we attempt to post-process ventricle masks first by a large closing element to remove any noises or dispersed punctuate regions, and then by a rather large dilation element like a rectangular 9-by-9 element multiple times, causing the mask dilated more than 10 mm in real-world terms to cover any problematic regions of unwanted GM.

#### 2.3.3 Gray matter processing

Our proposed method involves utilizing all three post-processed main brain tissue masks along with the WMH and ventricular masks, to filter out all unrelated, unwanted, and/or falsely labeled tissue that might be present in the GM map originally generated by the SPM. To achieve this, we create a union mask by combining the post-processed masks for WM, CSF, WMH, and ventricular masks. We then subtract binarily the post-processed GM mask from the union mask so that we obtain a specialized GM mask. The entire process of our proposed pipeline is illustrated and briefly described in Figure 3.

#### 2.3.4 cGAN training

**Figure 4:**
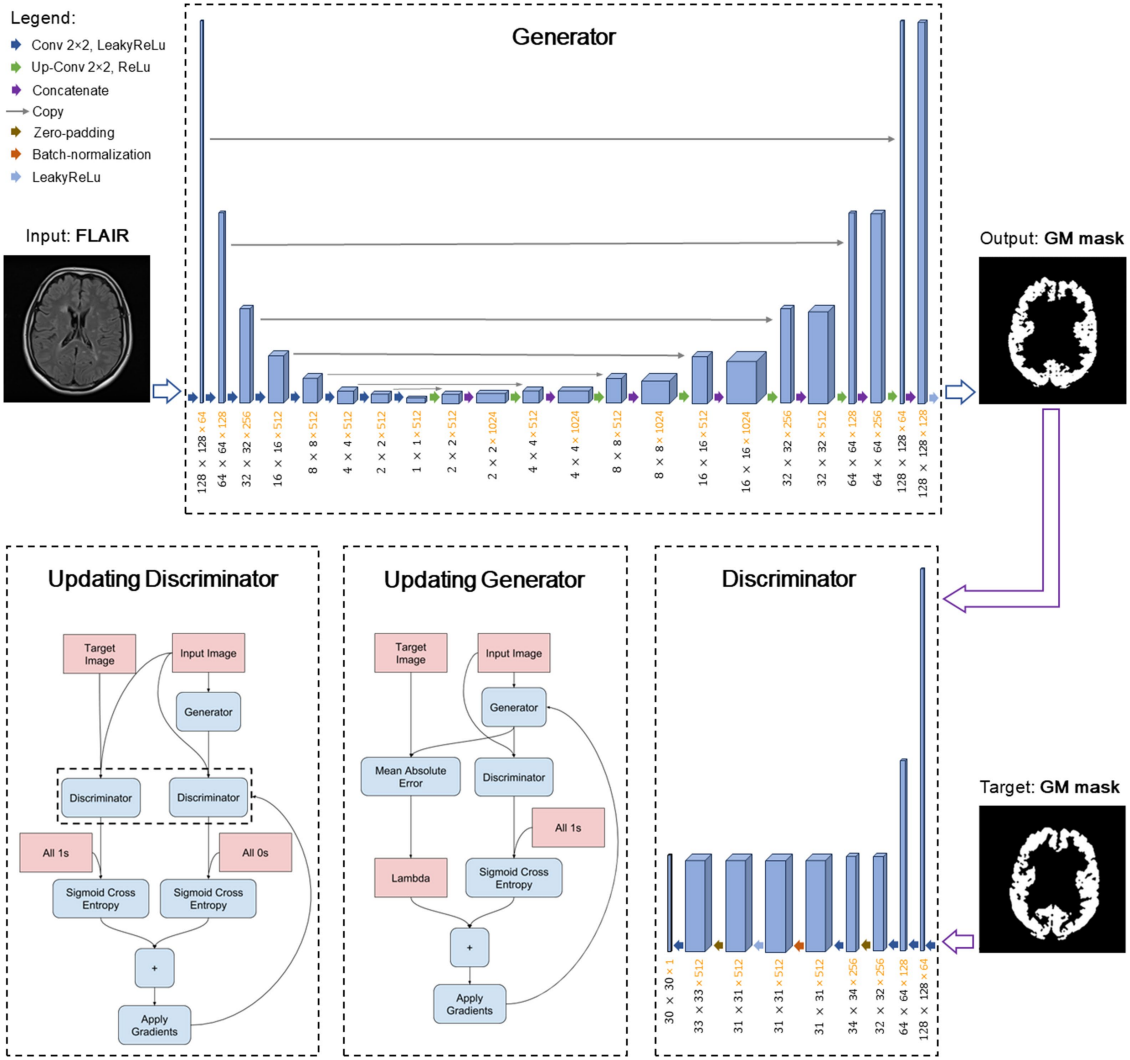
The architecture of pix2pix model. The model is trained with a paired set of images as input FLAIR and target GM mask. Updating blocks in the bottom row describe the learning process of corresponding networks.

As the last step of our proposed method, shown in Figure 3, we aim for training a cGAN for substituting the entire pipeline in generating GM masks. For our local dataset, which contains both healthy and non-healthy patients, we can utilize the pipeline to produce specialized GM masks. Subsequently, we can train a deep-learning model, such as the pix2pix model (Isola et al., 2016), also referred to as a cGAN, to learn the mapping between the input FLAIR images and the corresponding specialized GM masks. We use the data of the mentioned 9 MS patients, as validation data and split the rest into 80% for training and 20% for testing. The architecture of the model remains mostly unchanged as originally introduced by Isola et al. (2016). Figure 4 illustrates the structure of the pix2pix model, where the generator and discriminator units are a modified U-net network (Ronneberger et al., 2015) and a convolutional PatchGAN classifier (Isola et al., 2016), respectively. The generator network’s input and output are single images, while the discriminator’s input consists of the concatenation of the generated mask and the target mask. The discriminator’s output, in turn, provides a patch that contributes to the updating processes of both networks. The details of these updating processes are described separately in the bottom line of Figure 4.

### 2.4 WMH classification

In this study, as stated in the introduction, our objective is to classify WMH into three classes: periventricular, paraventricular, and juxtacortical. Therefore, to achieve this classification, we require both the shortest distance to the GM and ventricular masks. Initially, we obtain the contours of the GM and ventricular masks separately. Subsequently, for each WMH object in a given segmented image, we determine the object’s contour and calculate its distance to the ventricles’ contours. To do this, we choose the minimum measured Euclidean distance between the mass center of the object’s contour and each point on the ventricles’ contours. Additionally, we calculate the shortest distance between the object’s contour and the GM’s contours by selecting the minimum measured Euclidean distance between each point on the object’s contour and the GM’s contours. According to Algorithm 1, the understudy object will be classified as a periventricular WMH, if the shortest distance to the ventricles is equal to or less than 5 mm or if the distance of mass centers to the ventricles is equal to or less than 10 mm. Otherwise, it is classified as either paraventricular or juxtacortical. If the shortest distance to the GM mask is 5 mm or less and the area of the object is less than 20 mm^2^, the object is classified as juxtacortical. Otherwise, it is classified as paraventricular.

## 3 Results

### 3.1 GM segmentation

The enhancement of WMH classification relies on the accurate segmentation of ventricles and GM. Initially, three brain tissue masks, in addition to WMH and ventricle masks, were acquired for a patient. Subsequent post-processing steps were implemented to prepare these masks for integration into the final filtered GM mask. Finally, a deep learning model was trained to learn the entire process of specialized GM segmentation from FLAIR images only.

**Figure 5:**
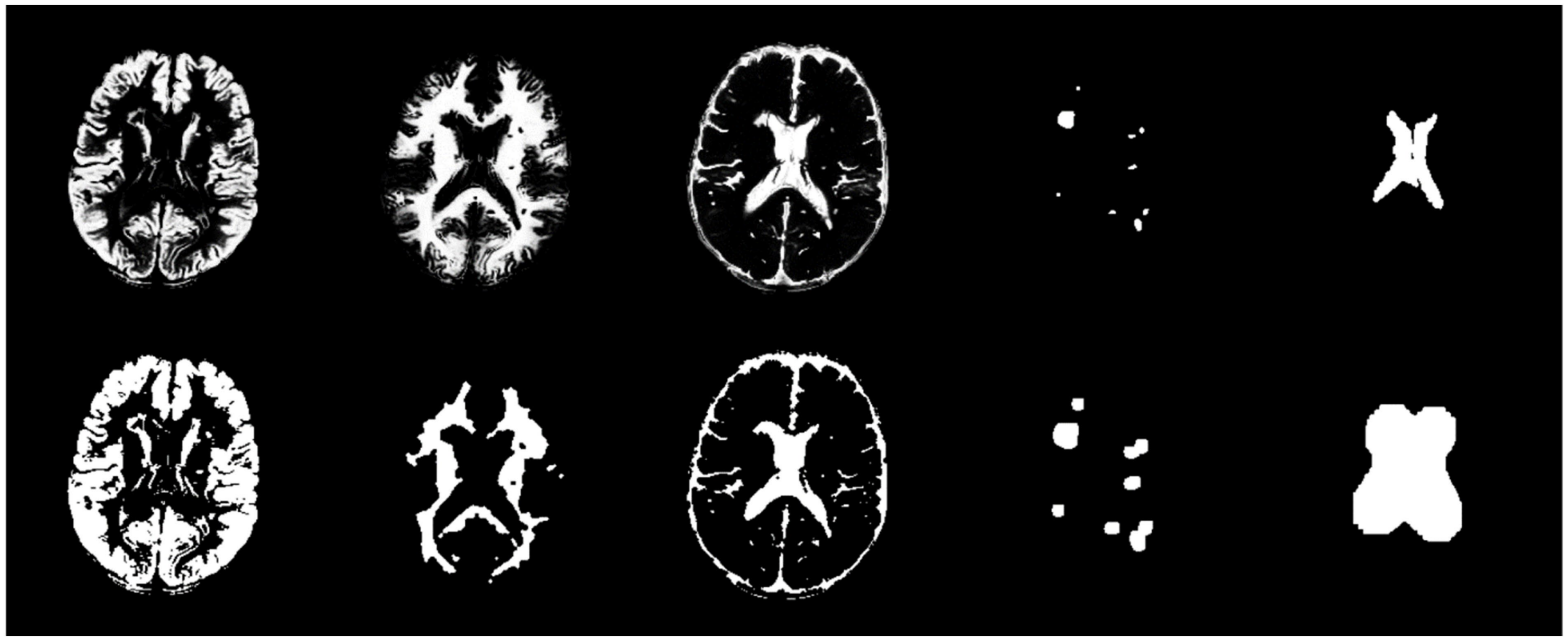
From left to right, the first row exhibits GM, WM, CSF, WMH, and ventricle masks, respectively. The second row exhibits post-processed masks by morphological operations for the corresponding masks in the first row.

In the method section, the SPM toolbox was employed to extract brain tissue maps from T1 images registered on FLAIR images. Furthermore, automatic algorithms such as LST-LPA and atlas-matching were utilized to provide WMH and ventricle masks, respectively. Figure 5 displays five extracted masks and their post-processing results. It is noteworthy that the first three images in the first row of Figure 5 are grayscale images, while the remaining images are binary.

**Figure 6:**
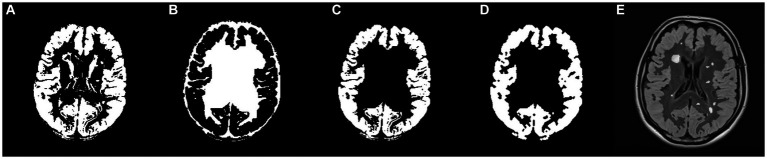
**(A)** Post-processed GM mask, **(B)** the union mask generated from WM, CSF, WMH, and ventricles masks, **(C)** filtered GM mask by the union mask, **(D)** post-processed, filtered GM mask, and **(E)** FLAIR image with the imposition of the generated GM mask and highlighted WMH.

Following the preparation of the five masks, the final GM mask was formulated. As illustrated in Figure 5, a union mask was initially created from the post-processed WM, CSF, WMH, and ventricle masks, which was then binarily subtracted from the post-processed GM mask. Eventually, the resulting GM mask was post-processed by basic morphological operations. Figure 6 shows the final GM mask, which serves as a specialized GM mask for JCWMH, and its transparent overlay on the corresponding FLAIR image of a patient.

### 3.2 cGAN training

To evaluate the performance of the trained cGAN model statistically, several metrics were used including accuracy, sensitivity [i.e., true positive rate (TPR)], and specificity [i.e., true negative rate (TNR)], as defined in Equations 1–3:


(1) $$\mathrm{Accuracy}=\frac{TP+TN}{TP+FN+TN+FP}$$

(2) $$\mathrm{Sensitivity}\;\mathrm{or}\;\mathrm{Recall}=\frac{TP}{TP+FN}$$

(3) $$\mathrm{Specificity}=\frac{TN}{TN+FP}$$

TP, TN, FP, and FN are short for true positive, true negative, false positive, and false negative, respectively. Additionally, as of the most commonly used metrics in medical image segmentation, precision, the Dice similarity coefficient (DSC), and intersection over union (IoU) were measured by subsequent Equations 4–6, respectively.


(4) $$\mathrm{Precision}=\frac{TP}{TP+FP}$$

(5) $$DSC=\frac{2\;TP}{2TP+FP+FN}$$

(6) $$IoU=\frac{TP}{TP+FP+FN}$$

Precision, Equation 4, measures a method’s ability to correctly identify only the relevant areas (true positives) without labeling irrelevant areas as positive (false positives). Sensitivity, Equation 2, focuses on the ability to capture all relevant instances, i.e., minimizing the number of false negatives. While, DSC, Equation 5, is a measure of how well the predicted segmentation overlaps with the ground truth. DSC is particularly useful because it balances the need for both high sensitivity (capturing most of the target region) and high precision (minimizing false positives). In practice, these metrics are often used together to provide a comprehensive evaluation of a segmentation method’s performance.

**Figure 7:**
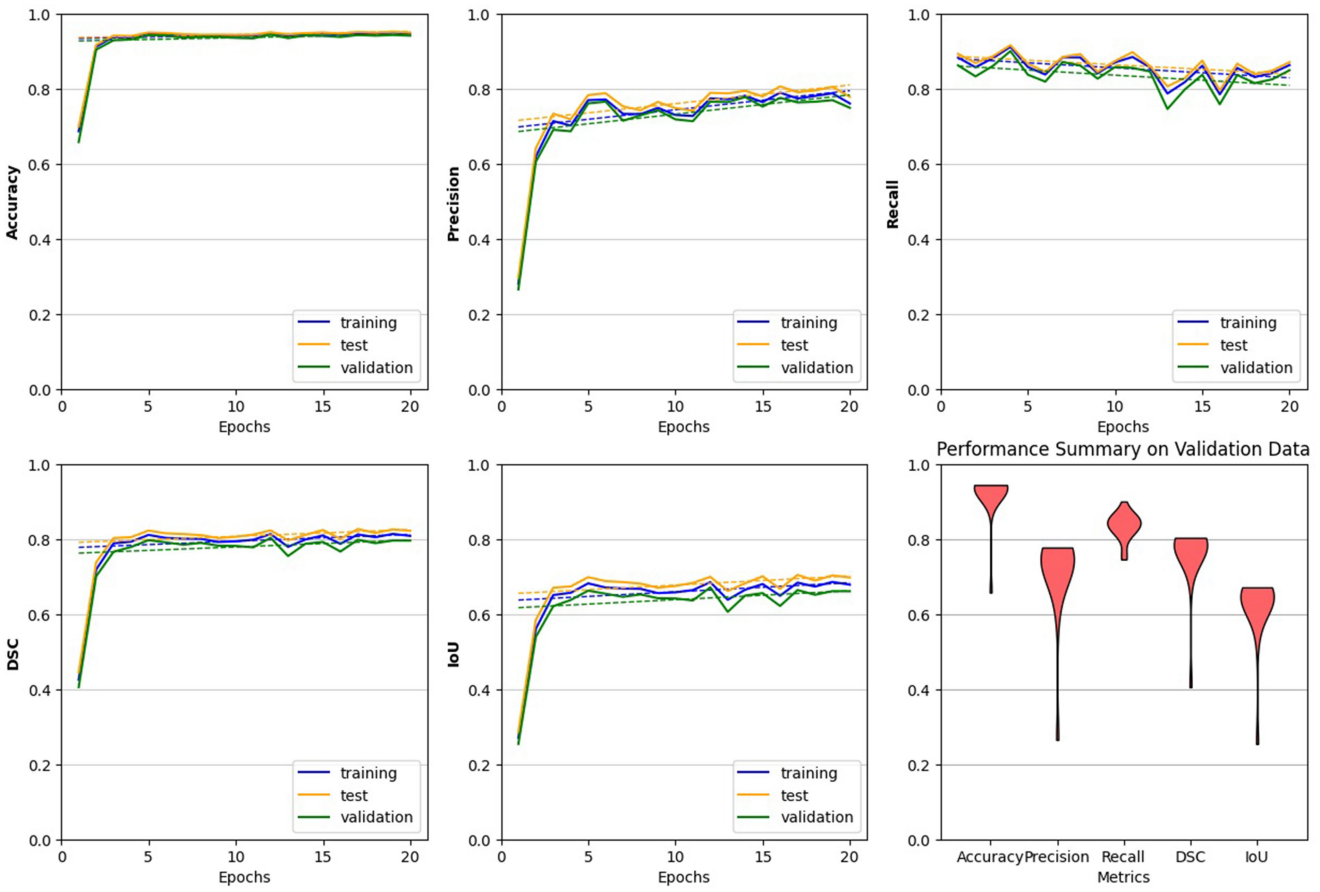
Performance evaluation of the trained pix2pix model. The model behaves more steadily after the 4th epoch, indicating a reliable choice for the trained model. Across all plots, with the exception of the final one, we analyzed the model’s performance on all three datasets. The last plot, represented by a violin plot, exclusively displays the model’s performance on the validation data across all five metrics.

**Figure 8:**
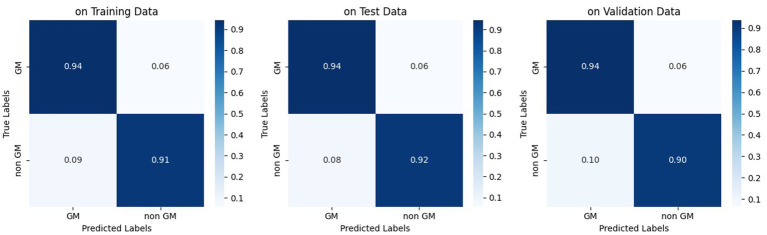
Normalized confusion matrix of the pix2pix model on fourth epoch. Figures from left to right, display the confusion matrix using training, test, and validation dataset, respectively.

**Figure 9:**
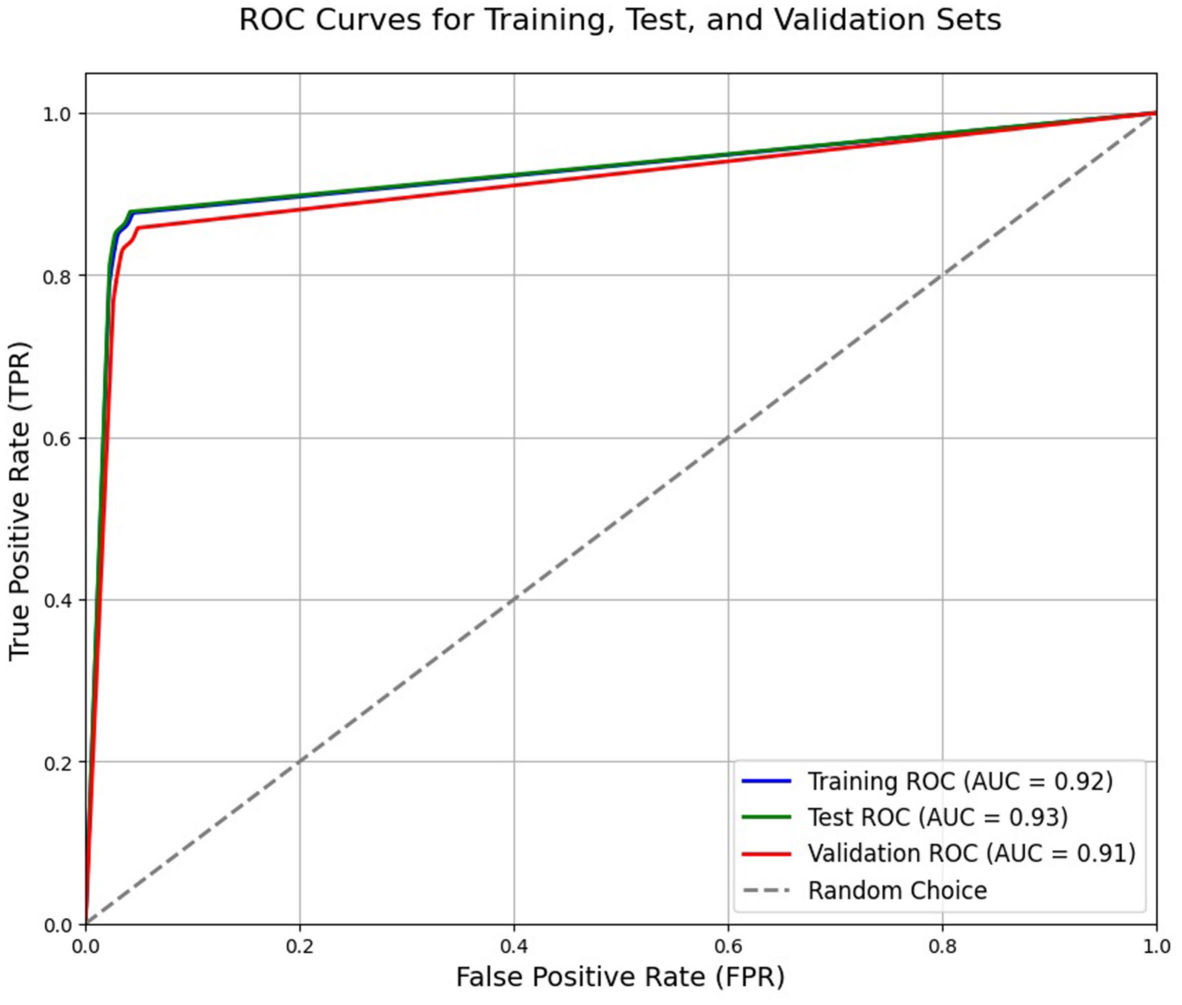
Receiver operating characteristic (ROC) curve of the trained model based on the fourth epoch. The reported AUC in the plot refers to area under the curve.

The study utilized all defined metrics except specificity to evaluate the performance of the trained model. The pix2pix model was trained on data from healthy individuals and MS patients for 20 epochs, as described in the method section. As shown in Figure 7, the model’s performance was observed to quickly improve from the first epoch to the fourth, after which it stabilized with only minor fluctuations. Both Figures 8, 9 demonstrate the stabilized and acceptable model performance in the fourth epoch. Examination of Table 1 provided further insights into the model’s performance at the fourth epoch and the average performance across all subsequent epochs. The comparison reveals that the model’s accuracy, DSC, and IoU were similar in both scenarios, with differences of about 1%. However, precision and recall differed by more than 5%, favoring one scenario for each metric. Therefore, it can be inferred that the fourth epoch represents a satisfactory point of peak learning for the model.

**Table 1:** Segmentation performance of the used pix2pix cGAN model at the 4th epoch and subsequent epochs.

Metrics	4th epoch			4th epoch till the end^a^		
	Training	Test	Validation	Training	Test	Validation
Accuracy	93.7	94	93.1	94.4 (0.33)	94.7 (0.32)	93.9 (0.37)
Precision	70.2	71.8	68.6	75.8 (2.45)	77.4 (2.45)	74.8 (2.55)
Recall	91.1	91.6	90	85.1 (3.25)	85.9 (3.05)	83.1 (3.71)
DSC	79.3	80.5	77.9	80.1 (0.96)	81.4 (0.91)	78.6 (1.18)
IoU	65.7	67.4	63.8	66.8 (1.33)	68.6 (1.29)	64.8 (1.58)

All numbers are in presented in percentage (%) format.

a Mean values and standard deviations are measured.

### 3.3 WMH classification

**Figure 10:**
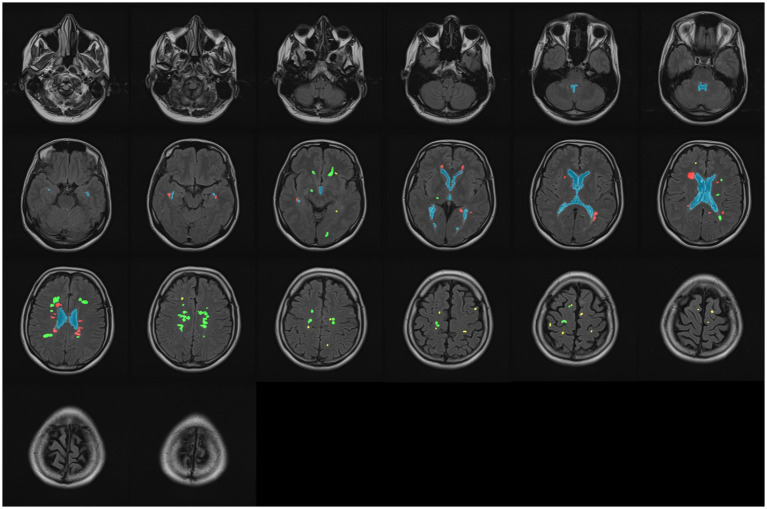
Classifying all WMH into three classes: periventricular (red), paraventricular (green), and juxtacortical (yellow). The ventricular system is marked by blue in all slices.

The final GM mask plays a crucial role in facilitating the intended WMH classification. In our study, Algorithm 1 was employed to determine the class of WMH detected in the FLAIR images. Additionally, specific colors were assigned to classes to enhance visual representation, as depicted in Figure 10. Alongside the visual presentation of the classified WMH, we conducted a statistical evaluation of the proposed method using various metrics mentioned in section 3.2, except for IoU.

To performance assessment, we calculated and presented five metrics in Tables 2, 3, listed in order of importance, based on the manual ground truth of data. Table 2 reports for utilizing the proposed pipeline without any training, while Table 3 reports for employing a trained model based on the pipeline, applied to the WMH classification task. The last column of both tables, labeled “Support,” indicates the actual number of pixels belonging to each WMH class. Also, the last two rows of the tables present simple and weighted averages for the aforementioned metrics.

**Table 2:** Classification report of using the proposed method—no training.

	DSC	Precision	Sensitivity	Specificity	Accuracy	Support
Periventricular	0.92	0.91	0.93	0.95	0.95	4,992
Paraventricular	0.91	0.92	0.90	0.91	0.90	8,040
Juxtacortical	0.78	0.75	0.80	0.97	0.95	1,450
Macro average	0.87	0.86	0.88	0.94	0.93	14,482
Weighted average	0.90	0.90	0.90	0.93	0.92	14,482

**Table 3:** Classification report of using the proposed method—with training.

	DSC	Precision	Sensitivity	Specificity	Accuracy	Support
Periventricular	0.92	0.91	0.93	0.95	0.95	4,992
Paraventricular	0.91	0.93	0.89	0.92	0.90	8,040
Juxtacortical	0.76	0.69	0.84	0.96	0.95	1,450
Macro average	0.86	0.84	0.89	0.94	0.93	14,482
Weighted average	0.90	0.90	0.90	0.93	0.92	14,482

### 3.4 Performance comparison

In order to compare our proposed method, we considered a commonly adopted approach in the literature for WMH classification. The most straightforward approach involves using T1 images to generate accurate GM masks, which are known for their high effectiveness. This approach solely relies on the gray matter mask extracted from registered T1 images onto FLAIR images, without any further processing (Jiménez-Balado et al., 2022).

**Figure 11:**
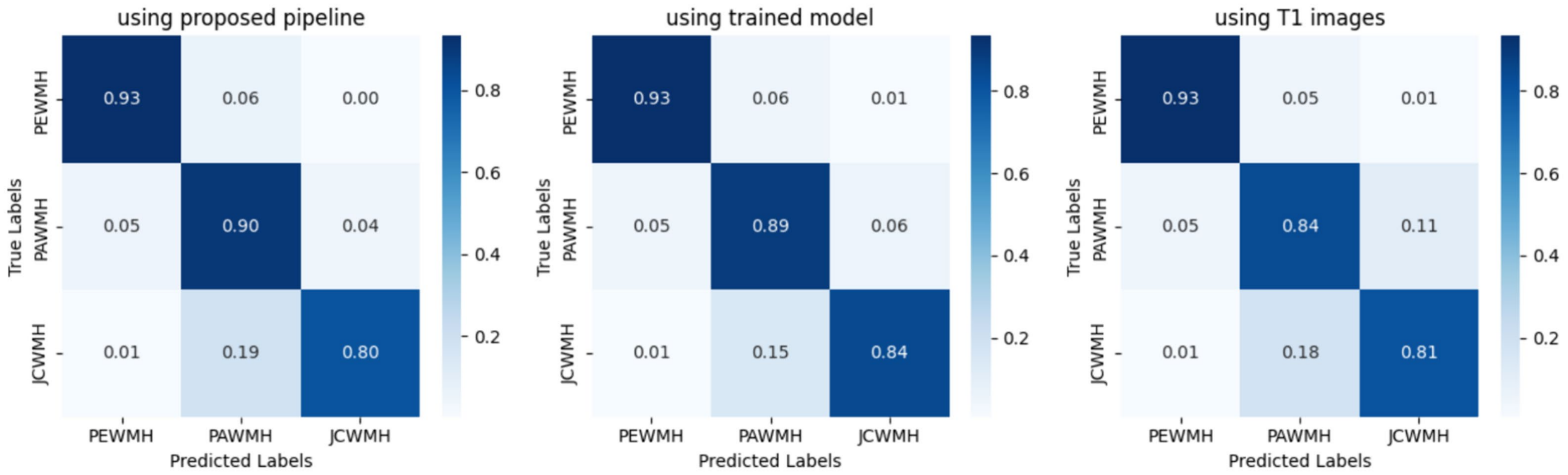
Normalized confusion matrix of classifying WMH. Figures from left to right, display confusion matrix using only the proposed pipeline, using trained model, and using only T1 images for the GM segmentation task, respectively.

Similar to our evaluation of the proposed method, we also conducted statistical analyses for this approach. It is worth noting that we initially registered T1 images to FLAIR images using the flirt command of FSL, and then segmented them using the SPM toolbox. Table 4 presents the classification results obtained by segmenting only the T1 images for GM masks. Moreover, we can see the origin of Tables 2–4 in Figure 11, which also demonstrates well the outperformance of WMH classification by using the trained model.

**Table 4:** Classification report of using T1 images segmentation.

	DSC	Precision	Sensitivity	Specificity	Accuracy	Support
Periventricular	0.92	0.91	0.93	0.95	0.95	4,992
Paraventricular	0.88	0.93	0.84	0.92	0.87	8,040
Juxtacortical	0.66	0.55	0.81	0.93	0.91	1,450
Macro average	0.82	0.80	0.86	0.93	0.91	14,482
Weighted average	0.87	0.89	0.87	0.93	0.90	14,482

### 3.5 Implementation details

The MATLAB programming language was utilized for brain tissue extraction in the proposed pipeline, whereas Python was employed for the remaining tasks, including classification. The implementation was carried out on a personal computer equipped with an Intel core-i7 CPU, an Nvidia RTX 3060 GPU, and 32G RAM running Windows10. Table 5 presents the implementation time for WMH classification using the proposed pipeline, both with and without training. It also includes the implementation time for using only T1 segmentation for GM masks in WMH classification. Given the wide range of WMH load in patients’ images and the substantial number of healthy individuals in our local dataset, we chose to report the rounded average execution time in Table 5. It is evident that the brain tissue segmentation (using SPM) and registration (using FSL or SPM) accounted for most of the execution time, while other parts of the pipeline were accomplished in less than a second. Furthermore, the classification task took less than 10 s, depending on the number of detected WMH. As a final note, only the GPU was utilized for training the model, while all other computations were performed on the CPU.

**Table 5:** Average execution time of producing a specialized GM mask for using the proposed method, in both with and without training, as well as using only T1 images segmentations.

	Proposed method no training	Proposed method with training	Only T1 segmentation
Training time	____	50 min^*^	____
**Execution time** ^**^	320 s	**<1 s**	110 s

* This was obtained using GPU.^**^More significant time due to the clinical practices.

## 4 Discussion

The diagnosis and monitoring of neurodegenerative brain diseases rely heavily on the detection and analysis of abnormalities in medical images. Automating the detection and classification of WMH can be immensely beneficial for both radiologists and neurologists. Computer-based methods for these tasks require relevant data, especially labeled data from MS patients. Unfortunately, there is currently no MRI dataset available that includes three or more class labels of WMH in MS. To address this gap, we collected data from healthy individuals and MS patients, and manually segmented and labeled some of them into PEWMH, PAWMH, JCWMH, and ventricular masks.

As outlined in Algorithm 1, PEWMH can be independently defined based on their distance from the ventricular masks, while PAWMH should be determined as neither PEWMH nor JCWMH. However, the main focus of this article is on distinguishing JCWMH from the other two classes. To support this classification task, we proposed a pipeline that incorporated a brain cortex map. As described in the method section, we have developed a process that ultimately generates a specialized gray matter mask, as shown in Figure 6. Although this mask may not precisely represent the segmentation of gray matter or cortex, it serves our purpose of classifying JCWMH most effectively.

The cGAN model was trained using the proposed pipeline to learn the entire process and generate a specialized GM mask solely from FLAIR images. The performance of the trained model was evaluated using the metrics presented in Table 1, demonstrating its effectiveness based on the ground truth provided by our proposed pipeline. However, it is important to note that the specialized GM masks produced by the pipeline, which served as the ground truth for model training, were not completely accurate and precise. Consequently, further investigation was conducted to assess the actual and practical performance of the model in the specific task of WMH classification, particularly for JCWMH.

Following the provision of a specialized GM mask through either the proposed pipeline or the trained model, WMH classification was carried out using Algorithm 1. Despite the limited manually segmented data, the proposed method demonstrated promising performance. Not only do the visual outcomes indicate that, but also the statistical metrics presented in Table 2 substantiate its competency. Precision and sensitivity, and the Dice similarity coefficient, which offers a harmonic mean between precision and sensitivity, are more decisive metrics for segmenting and classifying objects within an image. Hence, these significant numbers highlighted in Table 2, specifically pertain to the JCWMH classification performance. All three numbers support the success of the proposed method in JCWMH classification, though further refinements could enhance its effectiveness in the future. On the other hand, a comparison of the results obtained from the trained model, as presented in Table 3, indicates that the model performed on par with the proposed pipeline in overall WMH classification and achieved similar results in JCWMH classification. This highlights the superiority of the trained model due to its advantages, such as exclusively utilizing FLAIR images instead of T1 images and delivering results in less than a second. Therefore, we can employ the proposed pipeline once to generate sufficient paired training data for our model, and subsequently utilize the trained model to generate specialized GM masks.

Further elaborating on Tables 2, 3, the metrics of PEWMH classification were expected to reach 100% due to the manual WMH and ventricular masks provided. However, as indicated in Tables 2, 3, this did not happen. There are several arguments explaining this discrepancy. First, despite the use of manual segmentation, automatic methods lack the full extent of prior knowledge that experts have. For instance, due to slice thickness, ventricles might not be fully visible in an image, but an expert can mentally fill in the gaps, enabling them to correctly classify a WMH as PEWMH, whereas an automatic algorithm may struggle. Additionally, even though an expert performed manual segmentations for both ventricle and WMH tasks, there could be minor yet impactful errors, especially when making decisions near predefined limits or estimating the mass center of WMH. Last but not least, software errors such as rounding distances or making rigid choices near limits, can also contribute to lower PEWMH statistics. Unlike PEWMH, PAWMH is influenced by other classes, so improving the classification of JCWMH will naturally enhance the classification of PAWMH.

In order to evaluate the performance of the proposed method, Table 4 represents classification metrics when using only the segmentation of registered T1 images for GM masks. The metrics for PEWMH remained unchanged, as expected, since the ventricular masks remained the same. However, the classification results for JCWMH and consequently for PAWMH were notably lower. This was anticipated due to the inherent differences between T1 and FLAIR images, which are affected by artifacts and imperfections in registration algorithms. Also, the presence of some lesions may challenge and misguide GM segmentation even in high-contrast T1 images.

The proposed pipeline uses both T1 and FLAIR images and the obtained tissue masks from them to refine the primary GM mask obtained from T1 images. This pipeline may take longer time than common GM segmentation from T1 images, however, not only its outcome is much more reliable and useful for JCWMH classification, based on Tables 3, 4, but also it could provide paired data for training a subsequent deep model to replace itself. The trained model will generate the GM mask under a second for a new given data. Having examined the aforementioned approaches on WMH classification, the outperformance of the proposed method, particularly when using a trained model, is evident. The model, trained based on the proposed pipeline, offers several advantages, including automation, speed, no initials, and reliance on a single MR imaging weight. To ensure an unbiased and authentic evaluation of our proposed method, we employed manually segmented WMH and ventricles in this study. Moving forward, we could explore other advanced deep learning algorithms to achieve more precise segmentation of the cortex within FLAIR images, potentially surpassing our current method. Additionally, these algorithms could be employed to identify both simple and complex features of WMH, allowing for more meaningful classification through the analysis of extensive data on specific diseases. Furthermore, as a practical future direction, our automated method could be used to comprehensively study the longitudinal progression of neurodegenerative brain diseases, an emerging and crucial objective in clinical applications.

## 5 Conclusion

Our primary objective in this research was to classify WMH, particularly JCWMH, associated with neurodegenerative diseases like MS, into clinically interpretable categories to enhance the accuracy and inclusivity of brain disease diagnosis and monitoring analyses. Given the inadequacy of satisfactory classification methods and data, we first acquired a substantial local dataset containing manual segmentation and labeling for 9 MS patients. Subsequently, we proposed a pipeline to segment gray matter specialized for the classification of JCWMH. This pipeline was utilized to create paired data for training a cGAN model. Notably, the trained model relies exclusively on FLAIR images, despite the pipeline’s incorporation of both FLAIR and T1 images to generate specialized GM masks. The proposed method is automated, fast, and do not require any initials. To ensure unbiased and authentic evaluations, we employed data with manually segmented WMH and ventricles. DSC, precision, and sensitivity of our proposed method were 0.76, 0.69, and 0.84 for JCWMH and 0.90, 0.90, and 0.90 for all WMH, respectively. When compared to the common approach for extracting GM masks, using only registered T1 images on FLAIR images, our method demonstrated superior results, surpassing by a margin of at least 0.10 (i.e., 10%) in DSC. Needless to say, our method achieved these results using only FLAIR images and operates in less than a second, after being trained. Such cutting-edge methods will undoubtedly streamline the precise and meaningful analysis of WMH in clinical practice, reducing complexity and increasing efficiency.

## Data Availability

The original contributions presented in the study are included in the article/supplementary material, further inquiries can be directed to the corresponding author.

## Footnotes

^1^ https://www.mathworks.com/
